# Community/Crisis Cafés: Perspectives of Service Users and Carers Scoping Review

**DOI:** 10.1007/s10597-025-01484-7

**Published:** 2025-08-19

**Authors:** Neasa Ní Dhoibhilín, Owen Doody, David Bohan, Louise Murphy

**Affiliations:** 1 Health Service Executive, CHO7 Kildare West Wicklow Mental Health, Co.Kildare, Ireland; 2 School of Nursing and Midwifery, Faculty of Education and Health Sciences, University of Limerick, Limerick, Ireland; 3 Health Research Institute, University of Limerick, Limerick, Ireland; 4 Forum Connemara, Galway Road, Cliften, Galway, Ireland

**Keywords:** Crisis café, Community café, Mental health, Service-user, Peer support, Patient, Carer

## Abstract

Community/Crisis Cafés offer an alternative, out-of-hours mental health support by providing a safe, peer/clinician-supported environment for individuals in crisis. These cafés utilise peer support models that draw on personal experience, fostering connections and aiding in crisis management. Despite the growing global implementation of these cafés, limited research exists on how service users and carers experience and benefit from them. This study aims to explore the perspectives of service users and carers on accessing and utilising support through community/crisis cafés both nationally and internationally. A scoping review was guided by Arksey and O’Malley’s framework and included keyword searches of eight databases (Academic Search Complete, APA PsychInfo, CINAHL, Cochrane, Embase, Medline, Scopus, and Web of Science), combined with grey literature searches of LENSUS, Health Service Executive, WHO Global Index, NHS, and Open Grey. Backward and forward chaining of references was also completed to ensure all literature was sourced. Papers were limited to 2010–2023 and in English. Covidence was used for the screening process, ten papers met the review criteria and are reported as per the PRISMA-ScR checklist and PRISMA flow diagram. The findings of ten papers on service users and carers experiences indicate that Community/Crisis Cafés can have a positive impact on mental health management, alleviate social isolation, and reduce emergency department use. However, challenges such as consistency in service delivery and accessibility were noted. Further research and ongoing evaluation are necessary to fully understand the efficacy and limitations of this alternative co-produced, community mental health service delivery model.

## Background

The emergence of crisis or community cafés represents a shift in mental health service provision, offering out-of-hours, supportive spaces designed to provide immediate crisis prevention and response. These cafés, defined as safe, non-clinical environments typically open during evenings and weekends, aim to provide social, peer, and crisis support from trained professionals, volunteers, and peer workers (Butler & Hardiman, 2023). Although they are commonly referred to as crisis cafés, alternative names such as solace cafés, community cafés, or crisis centers are used interchangeably in the literature. This model has become increasingly recognised across countries as a response to the need for more accessible, community-based mental health support, particularly during times when traditional services are unavailable or overburdened (Collins, 2021; Workhouse Union, 2019). However, variations exist internationally regarding the implementation of community and crisis cafes and the service philosophy underpinning each (peer led versus clinician led). In the UK, for example, some crisis cafes are delivered as part of National Health Service (NHS) services and are clinician-led, not peer led.

The evolution of crisis/community cafés reflects a growing international acknowledgment that mental health crises require timely, compassionate responses that traditional services, such as emergency departments, may not always provide. The cafés originated as alternative mental health services that can operate during out-of-hours periods, reducing pressure on traditional emergency services and addressing people’s mental health care needs in a more tailored manner. Recent policy and advocacy efforts in countries such as Ireland, the United Kingdom (UK), and Australia emphasise the role of these cafés in mental health service delivery, particularly in expanding out-of-hours options for crisis intervention (Bateson et al., 2021; Consumers of Mental Health Western Australia, 2019).

Crisis cafés are distinct from conventional mental health services in that they are built on a foundation of peer support, which involves individuals drawing from their lived experiences of mental health challenges to assist others. These ‘lived experience’ workers also referred to in the literature as peer workers, pals, or allies utilise their own journeys through mental health distress, treatment, and recovery to provide support. Peer support is integral to fostering trust, understanding, and empowerment within these cafés, with benefits extending not only to service users but also to their families, carers, service providers, and the broader community (Bellamy et al., 2017; Stubbs et al., 2016). Crisis cafés serve as self-referral services, meaning individuals facing mental health challenges can access these spaces directly, without the need for a formal referral, offering a more accessible out-of-hours support option compared to emergency departments or community mental health teams (Healthwatch Wandsworth, 2019; Workhouse Union, 2019).

In Ireland, the Health Service Executive has included crisis cafés in its mental health service improvement plan for 2022–2024, recognising the need for alternative mental health support models (Health Service Executive, 2006). Among its recommendations, the Health Service Executive emphasises the importance of evaluating crisis cafés' effectiveness to ensure quality, best practices, and enhanced patient safety. This initiative advocates that these cafés should be developed in partnership with service users, carers, and professionals in a co-production model, defined as a collaborative process where public services are developed and delivered with equal input from both service users and providers (Health Service Executive, 2018). Additionally, “Sharing the Vision, A Mental Health Policy for Everyone” (Health Service Executive, 2020; Health Service Executive, 2022) supports the expansion of community and voluntary mental health services, emphasising reintegration and recovery for individuals experiencing mental health challenges. Crisis cafés align with this vision by providing individuals with a community-based alternative to hospital emergency departments, allowing them to address crises in a supportive, non-clinical environment.

The co-production approach in crisis cafés ensures that service users' perspectives are integral to service delivery, promoting a user-centered approach that respects individual strengths, resilience, and coping mechanisms. This aligns with Sláinte Care, Ireland’s health system reform program, which aims to provide community health services that enable individuals to access “the right care, in the right place, at the right time” (Health Service Executive, 2021a). The Health Service Executive’s recent development of a ‘crisis model of care’ echoes this ethos by proposing that crisis cafés be established nationwide, fostering a warm and welcoming atmosphere for those in distress and offering a community-centered alternative to emergency departments (Butler & Hardiman, 2023). Other countries, notably the UK, have also adopted crisis cafés as part of their mental health services, with the NHS finding that these settings provide compassionate and engaging support that can effectively address mental health crises outside regular hours (Wessex Academic Health Science Network, 2017).

The UK’s crisis cafés, also known as “safe havens” or “recovery cafés,” offer a non-clinical environment where individuals in mental health distress can find immediate, compassionate support. Reports indicate that in the absence of out-of-hours support, people facing mental health difficulties often turn to emergency departments (Wood, 2017). Crisis cafés mitigate this reliance by providing safe, accessible spaces for individuals to manage crises, prevent escalation, and reduce isolation. The settings focus on fostering an environment of respite and support, thus aiming to reduce emergency department usage while promoting early intervention. Core elements of effective crisis cafés include having peer supporters with lived experience and cultivating a welcoming, non-clinical atmosphere (Bateson et al., 2021; Collins, 2021). Peer supporters play a unique role by offering a sense of solidarity based on shared experiences, helping users feel understood and supported while also modeling that recovery is achievable.

Despite their promise, research on the impact of crisis/community cafés remains limited. Policies in Ireland, the UK, and Australia support these cafés’ implementation and advocate for service user and carer involvement in their development. However, only a few studies have rigorously examined how service users and carers experience and benefit from peer-supported crisis/community cafés. Notably, studies by Andrew et al., (2023) and Perkins et al., (2015) highlight the significance of peer support, with findings from Australia indicating that peer support roles within crisis cafés are pivotal for leadership and management. In addition, Staples et al., (2024) identified accessibility, being able to deliver person centered care, relationships with other services and staffing as factors influencing the effectiveness of crisis cafes. This underscores a need for further research to establish a more comprehensive understanding of crisis cafés’ impact on mental health service delivery and their role in supporting individuals in crisis.

The current scoping review aims to address this gap by exploring service users’ and carers’ perspectives on accessing and utilising support through crisis/community cafés. Given the paucity of well-conducted studies, particularly those involving direct feedback from services users and carers, this review seeks to contribute to the evidence base surrounding crisis/community cafés. By capturing the voices and experiences of those who use these cafés, this review aims to inform future policy and practice, ensuring that these community-based services effectively meet the needs of individuals in crisis and complement the broader mental health service landscape.

## Methods

To gain a comprehensive understanding of the experiences of service users and carers at community/crisis cafés, a scoping review was chosen as the most appropriate methodology. Scoping reviews allow for the broad exploration of existing literature on emerging topics and facilitate identification of gaps in knowledge (Arksey and O'Malley, 2005). Following Arksey and O’Malley’s six-step framework and adhering to PRISMA-ScR guidelines/flow diagram (Page et al., 2021; Tricco et al., 2018), this review mapped evidence on crisis cafés and peer-supported mental health services.

### Identifying the Research Question

Given the international variations in community/crisis cafes, a broad approach was used to include both forms of cafes (community and crisis cafes) within this review from both the service users and carers perspective. The primary research question was: What are the perspectives of service users and carers accessing community/crisis cafés? Using a PICo model to structure the question by population, intervention, and context, three sub-questions were formulated: (1) What evidence exists regarding service user involvement and co-production in crisis café development? (2) What are the perceived effects of crisis cafés? (3) How do service users and carers experience these cafés?

### Identifying Relevant Studies

A search strategy targeting both peer-reviewed and grey literature was developed (Table 1). Eight databases (Academic Search Complete, APA PsychInfo, CINAHL, Cochrane, Embase, Medline, Scopus, Web of Science) and four grey literature sources (LENSUS, Health Service Executive, NHS, Open Grey) were searched on 14th January 2024. To capture comprehensive results, backward and forward citation chaining was employed (reference lists and citations of included papers). The search strings for population, intervention, and context were searched separately using the Boolean operator OR in Title and Abstract and the search strings were combined using the Boolean operator AND (Table 2).

**Table 1:** Search terms table

Search terms		
Mental health OR Psychiatric OR mental ill health OR mental illness OR mental health problem OR mental health difficulties OR mental disorder	Customer OR service user OR client OR patient OR consumer OR family OR carer	safe haven* OR sanctuaries OR sanctuary OR crisis café OR crisis intervention OR peer support OR community-based center* OR community based center* OR Solace Café OR peer led OR wellness café OR connect café OR peer provider OR crisis café OR Solace Café OR wellness Café OR Connect Café

**Table 2:** Inclusion exclusion criteria

Inclusion	Exclusion
English language	Non-English language papers
Papers published from 01–01–2010 to 13–01–2024	Papers published before 01–01–2010
All types of literature	
Adult population	Child or adolescent population
Mental health	Non mental health setting or population
Crisis café, community-based café, peer-led café, wellness café, solace café, connect café, safe haven	Non-café based, non-community-based café

### Study Selection

**Fig. 1:**
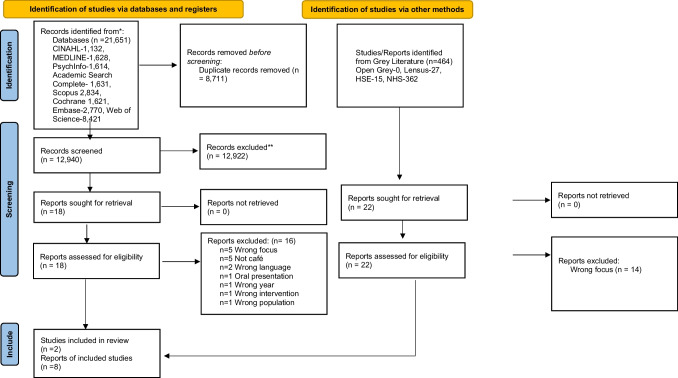
PRISMA Flow Diagram

From 21,651 records initially identified, 8,711 duplicates were removed. Titles and abstracts of 12,940 papers were screened using Covidence, resulting in 18 papers advancing to full-text review (Fig. 1). After rigorous assessment, two studies and eight grey literature reports met the inclusion criteria (Table 2). Screening was conducted by paired reviewers working independently with discrepancies resolved by a third reviewer to ensure methodological rigor.

### Charting the Data

Data from the included studies were charted to capture essential details, including author, year, title, country, aim or focus, design or approach, summary of findings, key messages and recommendations, limitations and type of service design (see Findings). This stage enabled categorisation of data by themes aligned with the three research questions, facilitating a systematic presentation of results.

### Collating and Summarising and Reporting the Data

The three questions set out in step one of the review processes were used to collate, synthesis and report the data. What is the evidence of service user involvement or co-production and peer involvement in the development of community/crisis cafés? What are the perceived effects of community/crisis cafés? What are the experiences of service users, and their families in accessing and using community/crisis cafés? Hong et al. (2018) mixed methods appraisal tool (MMAT) and Tyndall (2010) AACODS (Authority, Accuracy, Coverage, Objectivity, Date, Significance) appraisal tool for grey literature were utilised to assess the quality of included studies (Tables 3).

**Table 3:** Quality appraisal

Hong et al. (2018) Mixed methods appraisal tool (MMAT)		Andrew et al., (2023)	Bateson et al., (2021)	Collins (2021)	Consumers of Mental Health Western Australia (2019)	Health Service Executive (2022)	Healthwatch Wandsworth (2019)	Perkins et al., (2015)	Wessex Academic Health Science (2017)	Wood (2017)	Workhouse Union (2019)
Screening questions (for all types)	S1. Are there clear research questions?	Y						Y			
	S2. Do the collected data allow to address the research questions?	Y						Y			
	Further appraisal may not be feasible or appropriate when the answer is ‘No’ or ‘Can’t tell’ to one or both										
1. Qualitative	1.1. Is the qualitative approach appropriate to answer the research question?	Y						Y			
	1.2. Are the qualitative data collection methods adequate to address the research question?	Y						Y			
	1.3. Are the findings adequately derived from the data?	Y						Y			
	1.4. Is the interpretation of results sufficiently substantiated by data?	Y						Y			
	1.5. Is there coherence between qualitative data sources, collection, analysis and interpretation?	Y						Y			
2. Quantitative randomized controlled trials	2.1. Is randomization appropriately performed?										
	2.2. Are the groups comparable at baseline?										
	2.3. Are there complete outcome data?										
	2.4. Are outcome assessors blinded to the intervention provided?										
	2.5 Did the participants adhere to the assigned intervention?										
3. Quantitative nonrandomized	3.1. Are the participants representative of the target population?										
	3.2. Are measurements appropriate regarding both the outcome and intervention (or exposure)?										
	3.3. Are there complete outcome data?										
	3.4. Are the confounders accounted for in the design and analysis?										
	3.5. During the study period, is the intervention administered (or exposure occurred) as intended?										
4. Quantitative descriptive	4.1. Is the sampling strategy relevant to address the research question?										
	4.2. Is the sample representative of the target population?										
	4.3. Are the measurements appropriate?										
	4.4. Is the risk of nonresponse bias low?										
	4.5. Is the statistical analysis appropriate to answer the research question?										
5. Mixed methods	5.1. Is there an adequate rationale for using a mixed methods design to address the research question?										
	5.2. Are the different components of the study effectively integrated to answer the research question?										
	5.3. Are the outputs of the integration of qualitative and quantitative components adequately interpreted?										
	5.4. Are divergences and inconsistencies between quantitative and qualitative results adequately addressed?										
	5.5. Do the different components of the study adhere to the quality criteria of each tradition of the methods involved?										
Tyndall (2010) AACODS checklist for appraising grey literature											
Authority	Identifying who is responsible for the intellectual content **Individual author:** • Associated with a reputable organisation? • Professional qualifications or considerable experience? • Produced/published other work (grey/black) in the field? • Recognised expert, identified in other sources? • Cited by others? (use Google Scholar as a quick check) • Higher degree student under “expert” supervision? **Organisation or group**: • Is the organisation reputable? (e.g. W.H.O) • Is the organisation an authority in the field? **In all cases:** • Does the item have a detailed reference list or bibliography?		Y	Y	Y	Y	Y		Y	Y	Y
Accuracy	Does the item have a clearly stated aim or brief? • Is so, is this met? • Does it have a stated methodology? • If so, is it adhered to? • Has it been peer-reviewed? • Has it been edited by a reputable authority? • Supported by authoritative, documented references or credible sources? • Is it representative of work in the field? • If No, is it a valid counterbalance? • Is any data collection explicit and appropriate for the research? • If item is secondary material (e.g. a policy brief of a technical report) refer to • the original. Is it an accurate, unbiased interpretation or analysis?		Y	Y	Y	Y	Y		Y	Y	Y
Coverage	All items have parameters which define their content coverage. These limits might mean that a work refers to a particular population group, or that it excluded certain types of publication. A report could be designed to answer a particular question, or be based on statistics from a particular survey • Are any limits clearly stated?		Y	Y	Y	Y	Y		Y	Y	Y
Objectivity	It is important to identify bias, particularly if it is unstated or unacknowledged • Opinion, expert or otherwise, is still opinion: is the author’s standpoint clear? • Does the work seem to be balanced in presentation?		Y	Y	Y	Y	Y		Y	Y	Y
Date	For the item to inform your research, it needs to have a date that confirms relevance • Does the item have a clearly stated date related to content? No easily discernible date is a strong concern • If no date is given, but can be closely ascertained, is there a valid reason for its absence? • Check the bibliography: have key contemporary material been included?		Y	Y	Y	Y	Y		Y	Y	Y
Significance	This is a value judgment of the item, in the context of the relevant research area • Is the item meaningful? (this incorporates feasibility, utility and relevance) • Does it add context? • Does it enrich or add something unique to the research? • Does it strengthen or refute a current position? • Would the research area be lesser without it? • Is it integral, representative, typical? • Does it have impact? (in the sense of influencing the work or behaviour of others)		Y	Y	Y	Y	Y		Y	Y	Y

### Consultation Exercise

In alignment with Arksey and O’Malley’s optional consultation stage, a public patient involvement (PPI) consultation was conducted with Mr. David Bohan. His insights enriched the findings by providing a user-centered perspective that enhanced the quality and relevance of the review.

## Findings

### Study Characteristics

The review included ten sources: two qualitative studies and eight reports (see Table 4—Data Extraction). These comprised studies from Ireland (*n* = 3), the United Kingdom (*n* = 4), and Australia (*n* = 1), with sample sizes ranging from 7 to 106 participants (Andrew et al., 2023; Consumers of Mental Health Western Australia, 2019). The studies, published between 2015 and 2023, included service user involvement in four papers, while six involved both service users and carers or other stakeholders.

**Table 4:** Data extraction table

Author’s/Year/Title Country	Aim/Focus	Design: Methods/Methodology	Summary of Evidence	Key Messages/ Recommendations	Limitations	Involvement
Andrew et al. (2023) What makes a space safe: Consumers’ perspectives on mental health Australia	To gain an understanding of what mental health consumers think a safe space should look like	**Design:** Qualitative descriptive approach **Sample:** All participants (*n* = 7) had lived experience of mental health difficulties and/or crises and had attended an ED during a mental health crisis within the past 5 years **Data Collection:** Two 2-h Focus Groups utilising the COREQ checklist for interviews and focus groups method, 4 participants in one focus group and 3 participants in the second focus group **Data Analysis:** Inductive Thematic Analysis	Practical ideas of running a safe space were learned. There is a clear need of alternative supports to ED. Recognised some of the challenges in setting up a safe space Reduction in the number of ED attendances and police callouts Consumers were able to maintain autonomy and found experiences positive	Importance of physical and social features for a safe space that is inclusive and accessible that provides a sense of belonging and support provided by those with lived experience	Number of participants involved—6 Local geographical context	Presents service user perspectives on services
Bateson et al. (2021) Review of mental health services in Northern Ireland United Kingdom	To gather and evaluate the available evidence and perspectives in order to make recommendations for mental health crisis services	**Design:** Mixed methods report including: literature review; in depth meetings with wide range of services, and a co-produced online survey **Sample:** Total sample size unspecified. Representation from 4 key groups including policy leads for mental health services, service providers, service users and carers, and interagency partners and others **Data Collection:** Online in-depth meetings using a semi structured interview **Data Analysis:** Thematic analysis and Meta Analysis	The need for safe and accessible alternatives to ED for people in crisis. Is a concept that supports connectedness and brings crisis supports as an alternative to ED There is a lack of integration between café and statutory services	Co-production should be essential Recommends the piloting and evaluation of crisis cafés as well as safe spaces as alternatives to ED The environment plays a vital role in treatment options Lack of privacy in ED is not conducive to the biopsychosocial approach	Conducted in the context of COID 19 Timeframe in which review was conducted	Included service users perspectives on services
Collins (2021) Galway Community Café: Pilot study external evaluation Ireland	To provide an independent analysis of outputs and outcomes from the café	**Design:** Cross sectional quantitative pilot design **Sample Size:** Participants had lived experience of mental health challenges who attended the café as a customer (*n* = 13) **Data Collection:** Customer satisfaction survey (*n* = 13), weekly team reports (*n* = 15), and customer attendance logs (*n* = 11). Client experience and satisfaction were explored through an online survey and one-to-one feedback **Data Analysis:** Descriptive analysis of customers responses to satisfaction survey and quantitative analysis of monitoring data e.g. attendance trends	One of the recommendations of Sharing the Vision. Designed and delivered by people with lived experiences in coproduction Provides a space to talk to someone and to be kept safe. There is evidence of repeat attendees to the café There was a preference for face-to-face support vs phone or social media	Safe non-judgmental place for people to chat Strict protocols in place for peer supporters	COVID 19 limited how feedback could be given	Co production COVID 19 impacted the running of café
Consumers of Mental Health WA (2019) Alternatives to Emergency Departments project report (2019) Australia	Listening to and understanding the voices of people in Western Australia re: alternatives to Eds	**Design—**Qualitative, participatory research, co-designed with individuals possessing lived experience of mental health crises **Sample—**Individuals with lived experience of mental health crises, including those who have accessed emergency departments. Not explicitly stated **Data Collection—**Focus groups and interviews with consumers. Community consultations and workshops. Surveys and questionnaires to gather broader input. Collaboration with peer support networks and mental health organisations **Data Analysis—**Thematic analysis of qualitative data	Increased social networking. Diverting approx. 10 visits to ED per month based on ED data and up to 30 per month based on café data. Cost benefit in the utilisation of the café vs ED. Increased confidence for service users to pursue education/employment/ housing. Respect and peer support are highly valued Peer supporters effectively engage with clients	Need for alternative to ED waiting room that functions as a café Option to engage with peer support workers Should be open out of hours. Psychological support in social setting	Dos not provide a biopsychosocial support Do not provide acute care Better understanding is needed to meet the needs of individuals with intellectual disabilities along with mental health difficulties	Co-production – consumer, family member, carer, friend, service provider and stakeholders.
Health Service Executive (2022) Bridging and linkage to next appropriate care Ireland	To look at Crisis cafés as psychosocial crisis supports	**Design—**Clinical practice guideline **Sample—**Individuals presenting to Emergency Departments (EDs) or Suicide Crisis Assessment Nurse (SCAN) services with self-harm or suicidal ideation. Not specified **Data Collection—**Documentation of patient presentations and referrals. Maintenance of records for all ED and SCAN service referrals. Communication logs detailing follow-up calls and interactions with patients and General Practitioners (GPs) **Data Analysis—**Not explicit but includes mechanisms for monitoring and evaluating the implementation of guidelines	Crisis café have been identified as offering support in a number of countries. They provide an extra support alongside mental health statutory resources	Crisis cafés provide psychological support but lack the biopsychosocial approach	Does not provide a biopsychosocial approach	Peer led and non-clinical approach
Healthwatch Wandsworth (2019) Experiences of Wandsworth mental health recovery cafés United Kingdom	To gain a knowledge of peoples experience of recovery cafés	**Design**—mixed-methods evaluation using structured surveys and qualitative feedback **Sample**—total 47 people, 29 participants were users of the Recovery Café, 18 participants were individuals with mental health issues attending community groups but not the café. Majority female (18 of 29 café users). Diverse ethnic and cultural backgrounds. Ages mostly over 30; only 2 participants under 30 **Data Collection**—Two structured surveys/questionnaires conducted in-person, with researchers visiting the café and community groups **Data Analysis**—Descriptive statistics (e.g., frequencies of service use, number of crises, service rating). Thematic synthesis of open-text responses and quotes to highlight user perspectives, sentiments, and perceived barriers	23/29 found it helpful and a vital service for their mental health needs and support. Knowledge and friendliness of staff Stops a crisis happening An ability to talk in safe space	Need for more locations and longer hours. Use of café is needed to be clearer, only during a crisis or if could be used to avoid a crisis	Location and opening time	Service user experience
Perkins et al. (2015) Real lives: promoting recovery through personalisation and peer support United Kingdom	To describe the development of a community project providing peer support	**Design** – Qualitative, descriptive case study **Sample**—48 clients aged 18 to 95, 18 peers and allies for living aged 18 to 60 **Data Collection** – interviews **Data Analysis**—no detailed explanation of formal data analysis techniques, the paper appears to rely on narrative synthesis, using direct quotes and descriptive reporting to convey findings and reflections	It is possible to use peer support in the community to provide a service for people with mental health difficulties It allows for community connectedness	The need for the expertise of lived experience in providing a service within mental health There was a need for a safe space to run café Support via the lived experience has shown some success	There were no other similar services in the area Relied on word of mouth to gain clients attending Took time to ensure peer supporters were able to start peer work	Café manager and peer perspectives
Wessex Academic Health Science Network (2017) Aldershot Safe Haven Service United Kingdom	To evaluate and provide robust evidence of the impact of the Safe Haven service on service user experience	**Design**—Mixed-methods independent evaluation **Sample**—Cohort for ED analysis: 92 service users who had onward referrals from Safe Haven to NHS Trust **Sample**—79 respondents (via iPad survey), 369 respondents (via earlier paper-based questionnaire). Overall Attendance 4,275 attendances by 670 unique users. ED Impact Analysis: 92 individuals. Section 136/Police Data: Aggregated data from local policing districts and trust records **Data Collection**—Emergency Department (ED) and psychiatric admission records. Mental health-related police callouts and deployments. Section 136 detentions. In-service attendance logs with categories: crisis, prevention, social. iPad-based surveys (33-question instrument). Paper questionnaires (8-question feedback). Open-ended responses grouped thematically **Data Analysis**—ED attendance pre/post Safe Haven attendance (cohort analysis over 12 months). Psychiatric admission trends (aggregate trend analysis over 4 years). Comparison of Section 136 detentions vs. wider regional/national trends. Economic modelling of cost avoidance (ED attendances, psychiatric bed days). Thematic analysis of survey open-text responses	Overall downward trend in ED attendance in area of crisis café along with psychiatric admissions and police callouts in the catchment area of crisis café Psych admission in the area has reduced since its setup. Helped service users. Coping in crisis Providing safe space to talk. Helps manage their condition. Specialist support. Somewhere safe to go. Positive well-being. Staying alive	Service users really value the service. To look at gaining primary care data. Designated catchment area, however, did not discriminate towards people outside the catchment area. Reduces social isolation. Encourages independent management of difficulties to prevent crisis	Sample size is small. Unable to link qualitative and quantitative results to observe any trends	Service user experience
Wood (2017) Proposal for an out of hours crisis café for North East Glasgow United Kingdom	To explore possible alternatives to ED and to make a case for Crisis Café	**Design**- Community-informed service development proposal. Co-produced and participatory, drawing on lived experience and stakeholder engagement **Sample**—Individuals with lived experience of mental health crises, community members, and local stakeholders. Not explicitly quantified **Data Collection**—Informal conversations and discussions with individuals experiencing mental health challenges. Engagement with community groups and local organisations. Review of existing crisis café models and relevant literature **Data Analysis**—Thematic analysis of qualitative feedback and insights gathered from participants	Creating an out of hours service would meet the needs of many when they find themselves in need When advertised the uptake of crisis café increased as an alternative to ED	To provide an out of hours face to face support for people experiencing mental health difficulties To utilise the lived experience. Need to feel listened to Informal, non-judgemental space	Limited in its scope	Manager, carer and service user experience
Workhouse Union (2019) Crisis Café Kilkenny feasibility study Ireland	Explores aspects of developing out of hour’s service	**Design**—Qualitative, exploratory feasibility study **Sample**—105 individuals, 39 organisations and community/voluntary groups **Data Collection**—16 Conversation Sessions with key stakeholders and mental health providers. 2 Site Visits: To Aldershot (UK) and Galway (Ireland) to study operational models. 1 Feedback Session: Post-site visit reflections. 2 Focus Groups: With members of TASK (Training and Support Kilkenny) and GROW (National community-based mental health organization). 2 Visioning Workshops: 52 participants explored core themes: crisis definitions, space and place, types of support, and governance models. Used interactive methods including small group activities, facilitated discussions, visual mapping (hexagonal notes), and plenary feedback **Data Analysis**—Qualitative thematic synthesis	There is a clear need for a non-clinical service and support for people with mental health difficulties	Need for developing a self-referral non-clinical out of hours support. Needs to be space and place to set up café. Will rely on a governance structure	Location and funding	Co-production

The scoping review identified several key themes from the literature regarding community/crisis cafés and their role in supporting service users and carers in mental health crises. These included accessibility and value of crisis cafés, service users highly value accessible, and community-based support during crises, particularly when it includes peer support in an out-of-hours, café-style setting (Andrew et al., 2023; Bateson et al., 2021; Healthwatch Wandsworth, 2019; Wessex Academic Health Science Network, 2017; Wood, 2017). When crisis cafés were accessible, they played a critical role in supporting people in crisis and preventing crises from escalating (Wessex Academic Health Science Network, 2017). Service users cited benefits such as a safe, inclusive space that reduces isolation, provides out-of-hours access, and fosters a sense of community support (Andrew et al., 2023; Consumers of Mental Health Western Australia, 2019; Healthwatch Wandsworth, 2019; Perkins et al., 2015; Workhouse Union, 2020). Additionally, involving service users in the design and delivery of these services was frequently highlighted as a priority (Bateson et al., 2021; Collins, 2021; Wood, 2017). The role of crisis cafés as alternatives to emergency departments, while crisis cafés are valuable in mental health crisis management, the Health Service Executive in Ireland views these cafés as a supplementary service to emergency departments rather than a direct alternative, as they primarily provide psychological support without a biopsychosocial approach. Despite this limitation, literature underscores the need for accessible, non-clinical alternatives to emergency departments for individuals in crisis (Bateson et al., 2021; Perkins et al., 2015; Healthwatch Wandsworth, 2019; Wood, 2017).

- ***Q1. What is the evidence of service user involvement or co-production and peer involvement in the development of community/crisis cafés?***

The literature supports the integration of service user input in developing community/crisis cafés. Involving service users at organisational, state, and national levels particularly in the design of non-clinical, out-of-hours services ensures that cafés meet the real-world needs of those experiencing mental health challenges (Bateson et al., 2021; Collins, 2021; Perkins et al., 2015). Peer involvement also fosters recovery-oriented practices, as individuals with lived experience serve as peer supporters (Collins, 2021; Perkins et al., 2015). However, data is limited regarding the extent of service user involvement across all stages of café design and implementation. Literature suggests that co-production, especially through peer support, enhances mental health recovery and has proven beneficial for service users, carers, and stakeholders (Andrew et al., 2023; Collins, 2021; Healthwatch Wandsworth, 2019; Perkins et el., 2015; Workhouse Union, 2019).

- ***Q2. What are the perceived effects of community/crisis cafés?***

The review highlights the positive impact of community/crisis cafés on service users and carers. Several studies reported reductions in emergency department attendance following engagement with crisis cafés in the UK (Andrews et al., 2023; Consumers of Mental Health Western Australia, 2019), resulting in both cost savings and reduced demand on emergency services (Consumers of Mental Health Western Australia, 2019; Wood, 2017). Additionally, there was a noted decrease in police callouts for individuals in distress (Andrew et al., 2023; Consumers of Mental Health Western Australia, 2019). Service users reported that the café environment, unlike emergency departments, offered them a more positive and autonomous experience (Andrews et al., 2023; Bateson et al., 2021; Consumers of Mental Health Western Australia, 2019). The growth of crisis cafés in the UK further underscores the need for such alternatives to traditional emergency services (Andrews et al., 2023; Wessex Academic Health Science Network, 2017; Bateson et al., 2021; Wood, 2017). Additionally, the cafés supported social engagement, helping reduce isolation and prevent crises from escalating (Consumers of Mental Health Western Australia, 2019; Healthwatch Wandsworth, 2019; Perkins et al., 2015; Wessex Academic Health Science Network, 2017).

- ***Q3. What are the experiences of service users, and their families in accessing and utilising community/crisis cafés?***

Overall, service users and carers reported positive experiences with community/crisis cafés, valuing them as supportive and non-clinical alternatives to emergency departments. Improvements in mental health and the ability to openly discuss difficulties with peer workers were common themes (Consumers of Mental Health Western Australia, 2019; Healthwatch Wandsworth, 2019; Perkins et al., 2015). Service users described feeling welcomed, respected, and autonomous in their care decisions within the café environment, which contrasted with their experiences in clinical settings (Andrews et al., 2023; Collins, 2021). For many, crisis cafés addressed isolation and fostered connectedness within the community, allowing them to engage with new people and build social networks (Bateson et al., 2021; Consumers of Mental Health Western Australia, 2019; Healthwatch Wandsworth, 2019; Perkins et al., 2015; Wessex Academic Health Science Network, 2017). Service users and carers expressed a preference for safe, friendly environments over clinical settings, citing a desire to avoid the intimidating aspects of hospital settings, such as sterile aesthetics and depersonalising atmospheres (Andrews et al., 2023; Collins, 2021). Cafés provided a welcoming, non-judgmental space where individuals felt supported by peers in a more comfortable, inclusive environment (Andrews et al., 2023; Collins, 2021; Consumers of Mental Health Western Australia, 2019; Wood, 2017). Furthermore, the cafés' ability to accommodate multiple users at once made them accessible and convenient resources for those in crisis (Collins, 2021; Consumers of Mental Health Western Australia, 2019; Wessex Academic Health Science Network, 2017).

## Discussion

This review highlights the purpose and perceived benefits of community/crisis cafés, focusing on the experiences of service users and carers. Most of the available data originates from reports (*n* = 8), reflecting a limited but emerging evidence base. Key findings emphasise the importance of co-production and peer involvement, the value of the café environment, and the positive impact of these services on users and carers.

Service users report that crisis cafés play a crucial role in managing mental health crises, providing a safe, supportive environment that addresses their needs outside of traditional hours. Attendees appreciate having a secure space where they can communicate freely, receive peer support, and participate in various activities, all facilitated by friendly and knowledgeable staff (Andrews et al., 2023; Bateson et al., 2021; Collins, 2021; Consumers of Mental Health Western Australia, 2019; Healthwatch Wandsworth, 2019). The accessibility, location, and hours of operation of these cafés are also significant, with service users advocating for extended hours and daily availability to meet demand (Healthwatch Wandsworth, 2019; Wood, 2017). The review reveals that crisis cafés provide essential support during acute mental health crises, offering an alternative to emergency departments (ED) by providing a safe, non-judgmental space to discuss challenges, engage in activities, and receive practical support (Andrew et al., 2023; Healthwatch Wandsworth, 2019). Many users find that these services offer greater autonomy over their care, in contrast to the often clinical and depersonalising atmosphere of EDs, which can feel disempowering and intimidating (Collins, 2021).

There is growing consensus, nationally and internationally, that service users and people with lived experience should be integral to all stages of mental health service development and delivery (Bateson et al., 2021; Health Service Executive, 2021b). Involving service users in policy and planning provides valuable insights that improve services by highlighting both successful elements and areas needing enhancement (Mental Health Commission, 2023; Australian Government, 2022). Crisis cafés have demonstrated the effectiveness of peer support, as individuals with lived experience provide empathetic assistance and foster a recovery-oriented practice (Collins, 2021; Perkins et al., 2015). Peer support is increasingly recognised as essential, with Australia’s Fifth National Mental Health and Suicide Plan underscoring the critical role of peer support workers (Department of Health, 2017). While crisis cafés are recognised as valuable mental health resources, they are not intended to replace EDs or comprehensive mental health teams. This is further reinforced by Staples et al (2024) who identified five perceived aims for crisis cafes including: provision of an alternative to EDs; improved accessibility to crisis care; provision of someone to talk to in a safe and comfortable space for people in acute distress; effective triaging; and improved crisis planning and people’s coping skills. The Health Service Executive in Ireland views crisis cafés as supplemental services that provide psychological support without employing a full biopsychosocial approach (Bateson et al., 2019; Health Service Executive, 2022). However, the NHS recognises that crisis cafés can effectively meet the needs of individuals in mental distress, often offering a more suitable alternative to EDs, which are not designed for ongoing mental health support (Wessex Academic Health Science Network, 2017).

Accessibility, both in terms of location and hours, is a vital aspect of crisis café utilisation. Studies highlight that service users greatly value the ease of access and proximity of these services, which reduce the need for travel, especially during a crisis (Cortina & Hardin, 2023; Staples et al., 2024). Service users report that having a nearby crisis café provides a “safe” place to go, fostering a sense of security and support (Collins, 2021; Wood, 2017). However, limited geographic availability remains a barrier, and many potential users advocate for more widespread, conveniently located cafés that are accessible to diverse populations (Bunyi et al., 2021). One notable impact of crisis cafés is the reduction in ED visits and mental health-related police callouts, as users are able to access immediate support in a non-clinical setting (Andrew et al., 2023; Consumers of Mental Health Western Australia, 2019). By providing a welcoming, non-judgmental environment, crisis cafés help prevent crises from escalating, which in turn alleviates pressure on emergency services. Despite these promising outcomes, there remains a scarcity of crisis cafés globally, and their presence is still relatively limited in many countries. In Ireland, recent policies recommend incorporating crisis cafés into the national mental health framework, but implementation remains in its early stages.

Despite the reported benefits, community/crisis cafés face several challenges, including limited availability, restricted hours, and insufficient integration into broader mental health services. Expanding these services and aligning them with community mental health teams could address some of these limitations. Additionally, incorporating a more comprehensive biopsychosocial approach may enhance the support provided in crisis cafés, enabling them to address a wider range of mental health needs. However, Staples et al (2024) identified a number of trade offs that mental health services should consider when designing and running a crisis café – balancing an open door policy with managing service demand through referral routes, balancing risk management procedure with the remit of offering a non-clinical environment, and raising community awareness while preventing stigma.To maximise the impact of crisis cafés, continued research and evaluation are essential. Expanding the evidence base with high-quality studies will provide deeper insights into the experiences of service users and carers, ultimately contributing to the development of best practices for these services. Future research should also explore long-term outcomes, such as sustained mental health improvement, user satisfaction, and system-level impacts on healthcare utilisation and costs.

The availability of peer-supported, out-of-hours crisis services for individuals with mental health challenges is essential for providing effective support during a crisis and helping prevent crises from developing. This article arrives at a time when community/crisis cafés are still in their early stages globally, advancing through policy initiatives and co-production models. This scoping review underscores the importance of understanding the experiences of service users and carers who utilise these cafés, particularly in shaping their development and delivery. From a mental health nursing and healthcare perspective, this review contributes valuable insights to the existing evidence base, enhancing mental health nurses’ knowledge of community/crisis cafés and their role in supporting clients. By familiarising themselves with these services, nurses and healthcare professionals can offer their clients and carers additional psychoeducation on resources available beyond primary mental health services. This information empowers clients to pursue recovery-oriented strategies in a safe, non-clinical, out-of-hours setting, offering an alternative to traditional crisis interventions.

### Limitations

The limitations of this scoping review primarily stem from the methodology used. Given the relatively new and evolving nature of community/crisis cafés, there is a limited body of data available. This review restricted research to publications from 2010 to 2023 to ensure relevance and currency, though it is possible that this criterion excluded potentially informative earlier studies. Additionally, only English-language papers were included, which may have limited the diversity of perspectives captured, especially from non-English-speaking regions where crisis cafés may be developing differently. Although quality appraisal is not typically required in scoping reviews, a quality assessment tool was applied to enhance the rigor and value of this review. However, it is important to recognise that scoping reviews inherently do not provide a comprehensive synthesis or critical analysis of study quality, which may limit the depth of conclusions that can be drawn.

## Conclusion

This review highlights the critical role of community/crisis cafés as accessible, supportive spaces for individuals in mental health crises. These cafés provide a valuable alternative to traditional services by allowing individuals to seek help in a secure, non-clinical environment. The involvement of peer support workers and a co-production model enhances these services, fostering shared experience and empowerment among service users. However, challenges such as limited availability and insufficient integration into the broader mental health system persist, indicating a need for further development and expansion. For individuals experiencing mental health difficulties, timely and appropriate care is essential. The literature suggests that peer-delivered services through crisis cafés show promise as a supportive strategy for those in crisis. However, additional research is necessary to better understand the perspectives of community/crisis café users and carers. This will be crucial to informing and refining the co-production and delivery of these services, especially given the current scarcity of information in this area. While the scope of this review is limited by the availability of literature, the insights gathered offer a valuable foundation for future research and service planning. The findings can guide researchers, policymakers, and service providers in promoting community-based mental health interventions that improve patient outcomes and reduce reliance on emergency services. By addressing the gaps identified, community/crisis cafés have the potential to become integral components of mental health support systems, positively impacting service users and carers alike.

## References

[CR1] Andrew, L., Karthigesu, S., Coall, D., Sim, M., Dare, J., and Boxall, K. (2023). What makes a space safe? Consumers’ perspectives on a mental health safe space. *International Journal of Mental Health Nursing*, 32(5), 1355–1364, available: 10.1111/inm.13174. Accessed 14 December 2023 10.1111/inm.13174 37231985

[CR2] Arksey, H., & O’Malley, L. (2005). Scoping studies: Towards a methodological framework. *International Journal of Social Research Methodology,**8*(1), 19–32. 10.1080/1364557032000119616

[CR3] Australia Government (2022). *2020–25 National Health Reform Agreement.* Australia, available: https://federalfinancialrelations.gov.au/sites/federalfinancialrelations.gov.au/files/2021-07/NHRA_2020-25_Addendum_consolidated.pdf. Accessed 16 December 2023

[CR4] Bateson, C., Allen, A., Cunningham, T., Davidson, G., McFeely, E., McGarry, P., & O’Connor, R. (2021). *Review of Mental Health Crisis Services in Northern Ireland*, Ireland: Queens University Belfast, available: https://pureadmin.qub.ac.uk/ws/portalfiles/portal/236583313/Review_of_mental_health_crisis_services_in_Northern_Ireland.pdf. Accessed 14 December 2023

[CR5] Bellamy, C., Schmutte, T., & Davidson, L. (2017). An update on the growing evidence base for peer support. *Mental Health and Social Inclusion,**21*(3), 161–167. 10.1108/MHSI-03-2017-0014

[CR6] Bunyi, J., Ringland, K. E., & Schueller, S. M. (2021). Accessibility and digital mental health: Considerations for more accessible and equitable mental health apps. *Frontiers in Digital Health,**3*, 742196–742196. 10.3389/fdgth.2021.742196 34713206 10.3389/fdgth.2021.742196 PMC8521906

[CR7] Butler, M. & Hardiman, S. (2023). *Crisis Resolution Services, Model of Care*, Ireland, Health Service, available: https://www.hse.ie/eng/services/list/4/mental-health-services/crs-moc.pdf. Accessed 14 Decemebr 2023

[CR8] Collins, K. (2021). *Galway Community Café Pilot Study External Evaluation,* Ireland: Dr Karina Collins Consultancy Service.

[CR9] Consumers of Mental Health Western Australia (2019). *Alternatives to Emergency Departments Project Report*, Australia: Consumers of Mental Health Australia, available: https://www.mhc.wa.gov.au/media/2993/alt-to-ed-and-safe-havens-final-report-2019.pdf. Accessed 14 Decemebr 2023

[CR10] Cortina, J., & Hardin, S. (2023). The geography of mental health, urbanicity, and affluence. *International Journal of Environmental Research and Public Health,**20*(8), 5440. 10.3390/ijerph20085440 37107722 10.3390/ijerph20085440 PMC10138034

[CR11] Department of Health (2017). *The fifth national Mental Health and Suicide Prevention Plan,* Australia, Commonwealth of Australia, available: https://www.mentalhealthcommission.gov.au/getmedia/0209d27b-1873-4245-b6e5-49e770084b81/Fifth-National-Mental-Health-and-Suicide-Prevention-Plan.pdf. Accessed 16 Decemebr 2023

[CR12] Health Service Executive (2006). A Vision for Change. Ireland, Government of Ireland.

[CR13] Health Service Executive (2018). *Co-production in Practice Guidance Document 2018–2020,* Ireland, Government of Ireland, available: https://www.hse.ie/eng/services/list/4/mental-health-services/advancingrecoveryireland/national-framework-for-recovery-in-mental-health/co-production-in-practice-guidance-document-2018-to-2020.pdf

[CR14] Health Service Executive (2020). *Sharing the Vision: A Mental Health Policy for Everyone,* Ireland, Government of Ireland, available: file:///C:/Users/neasanidhoibhilin/Downloads/76770_b142b216-f2ca-48e6-a551–79c208f1a247%20(3).pdf. Accessed 14 December 2023

[CR15] Health Service Executive (2021b). *HSE Corporate Plan 2021–2024,* Ireland, Health Service Executive, available at: https://assets.publications.hse.ie/media/file_based_publications/HSE_corporate_plan_2021-2024.pdf. Accessed 14 December 2023

[CR16] Health Service Executive (2021a). *Sláinte Care Implementation Strategy and Action Plan 2021–2023*, Ireland, Government of Ireland, available at: https://www.gov.ie/pdf/?file=https://assets.gov.ie/134746/9b3b6ae9-2d64-4f87-8748-cda27d3193f3.pdf#page=null. Accessed 14 December 2023

[CR17] Health Service Executive (2022). *Bridging and linkage to next appropriate care,* Ireland: Health Service Executive, available: https://www.hse.ie/eng/about/who/cspd/ncps/self-harm-suicide-related-ideation/moc/ncpsh-model-of-care-by-chapters/chap-6-bridging-and-linkage-to-next-appropriate-care.pdf. Accessed 14 December 2023

[CR18] Health Service Executive and Department of Health (2022). *Sharing the Vision Implementation Plan 2022–2024,* Ireland, Government of Ireland, available: https://www.drugsandalcohol.ie/35981/1/sharing-the-vision-implementation-plan-2022.pdf. Accessed 14 December 2023

[CR19] Healthwatch Wandsworth (2019). *Experiences of Wandsworth Mental Health Recovery Cafés*, United Kingdom: Healthwatch Wandsworth, available: https://www.healthwatchwandsworth.co.uk/sites/healthwatchwandsworth.co.uk/files/Final%20Report%20-%20HWW%20Recovery%20Café%20survey%20%28003%29.pdf. Accessed 14 Decemeber 2023

[CR20] Hong, Q.H., Pluye, P., Fábregues, S., Bartlett, G., Boardman, F., Cargo, M., Dagenais, P., Gagnon, M., Griffiths, F., Nicolau, B., O’Cathain,A., Rosseau, M & Vedel, I. (2018). *Mixed Methods Appraisal Tool (MMAT),* Version 2018, available: http://mixedmethodsappraisaltoolpublic.pbworks.com/w/file/fetch/127916259/MMAT_2018_criteria-manual_2018-08-01_ENG.pdf 10.1016/j.jclinepi.2019.03.008 30905698

[CR21] Mental Health Commission (2023). *Supporting Change Strategic Plan,* Ireland: Mental Health Commission, available: https://www.mhcirl.ie/sites/default/files/2023-04/10876%20MHC%20Strategic%20Plan%202023-2027%20COMPLETED.pdf. Accessed 16 Decemebr 2023

[CR22] Page, M.J., McKenzie, J.E., Bossuyt, P.M., Boutron, I., Hoffmann, T.C., Mulrow, C.D., Shamseer, L., Tetzlaff, J.M., Akl, E.A., Brennan, S.E., Chou, R., Glanville, J., Grimshaw, J.M., Hróbjartsson, A., Lalu, M.M., Li, T., Loder, E.W., Mayo-Wilson, E., McDonald, S., McGuinness, L.A., Stewart, L.A., Thomas, J., Tricco, A.C., Welch, V.A., Whiting, P., & Moher, D. (2021). The PRISMA 2020 statement: an updated guideline for reporting systematic reviews. *BMJ (Online)*, 372, n71–n71, available: 10.1136/bmj.n71 10.1136/bmj.n71 PMC8005924 33782057

[CR23] Perkins, R., Atkins, J., Hunter, N., Repper, P., Robertson, P.D., Thornton, P., and Thornton, S. (2015). Real lives: promoting recovery through personalisation and peer support. *Mental health and social inclusion*, 19(1), 22–29. available: 10.1108/MHSI-11-2014-0037. Accessed 14 Decemebr 2023

[CR24] Staples, H., Cadorna, G., Nykavaranda, P., Maconick, L., Llyod-Evans, B., & Johnson, S. (2024). A qualitative investigation of crisis cafes in England: Their role, implementation, and accessibility and accessibility. *BMC Health Services Research,**24*, 1319. 10.1186/s12913-024-11662-0 39478622 10.1186/s12913-024-11662-0 PMC11526642

[CR25] Stubbs, B., Williams, J., Shannon, J., Gaughran, F., and Craig, T. (2016). Peer support interventions seeking to improve physical health and lifestyle behaviours among people with serious mental illness: A systematic review. *International journal of mental health nursing*, 25(6), 484–495, available: 10.1111/inm.12256 10.1111/inm.12256 27600483

[CR26] Tricco, A. C., Lillie, E., Zarin, W., O’Brien, K. K., Colquhoun, H., Levac, D., Moher, D., Peters, M. D. J., Horsley, T., Weeks, L., Hempel, S., Akl, E. A., Chang, C., McGowan, J., Stewart, L. A., Hartling, L., Aldcroft, A., Wilson, M. G., Garritty, C., & Straus, S. E. (2018). PRISMA extension for scoping reviews (PRISMA-ScR): Checklist and explanation. *Annals of Internal Medicine,**169*(7), 467–473. 10.7326/M18-0850 30178033 10.7326/M18-0850

[CR27] Tyndall, J. (2010). *AACODS checklist*. Flinders University

[CR28] Wessex Academic Health Science Network. (2017). *Independent Evaluation of the North East Hamshire and Farnham Vanguard Aldershot Safe Haven Service,* United Kingdom: Wessex Academic Health Science Network, available: https://healthinnovationwessex.org.uk/img/projects/Safe%20Haven%20Evaluation_FINAL_October%202017_website.pdf. Accessed 14 Decemeber 2023

[CR29] Wood, C.E. (2017). *Proposal for an Out of Hours Crisis Café for North East Glasgow*, United Kingdom, available: https://www.purepotentialscotland.co.uk/wp-content/uploads/2017/10/Proposal-for-an-Out-of-Hours-Crisis-Cafe-NE-Glasgow-1ST.pdf. Accessed 14 Decemeber 2023

[CR30] Workhouse Union. (2019). *Crisis Café Kilkenny Feasibility Study, Ireland:* Workhouse Union, available: https://workhouseunion.com/wp-content/uploads/Crisis-Café-Kilkenny-Feasibility-Study-Online-Version.pdf. Accessed 14 December 2023

